# Predictors of Lassa fever diagnosis in suspected cases reporting to health facilities in Nigeria

**DOI:** 10.1038/s41598-023-33187-y

**Published:** 2023-04-21

**Authors:** Chinwe Lucia Ochu, Lorretta Ntoimo, Ikenna Onoh, Friday Okonofua, Martin Meremikwu, Sandra Mba, Akanimo Iniobong, Obinna Nwafor, Mahmood Dalhat, Cornelius Ohonsi, Chinedu Arinze, Ekpereonne Esu, Ehimario Uche Igumbor, Chioma Dan-Nwafor, Elsie Ilori, Ifedayo Adetifa

**Affiliations:** 1 Department of Prevention Programmes and Knowledge Management, Nigeria Centre for Disease Control, Abuja, Nigeria; 2 Department of Demography and Social Statistics, Faculty of Social Sciences, Federal University Oye-Ekiti, Oye-Ekiti, Nigeria; 3 Department of Health Emergency Preparedness and Response, Nigeria Centre for Disease Control, Abuja, Nigeria; 4 Centre of Excellence in Reproductive Health Innovation, University of Benin, Benin City, Nigeria; 5 Cochrane Nigeria, Institute of Tropical Diseases Research and Prevention, University of Calabar Teaching Hospital, Calabar, Nigeria; 6 Department of Paediatrics, University of Calabar Teaching Hospital, Calabar, Nigeria; 7 Department of Surveillance and Epidemiology, Nigeria Centre for Disease Control, Abuja, Nigeria; 8 Infectious Diseases Control Centre, Kaduna, Kaduna State Nigeria; 9 Department of Public Health, College of Medical Sciences, University of Calabar, Calabar, Nigeria; 10 Centre for Infectious Disease Research, Nigerian Institute of Medical Research, Lagos, Nigeria; 11 The Office of the Director General, Nigeria Centre for Disease Control, Abuja, Nigeria

**Keywords:** Diseases, Risk factors, Signs and symptoms

## Abstract

Lassa fever (LF) remains endemic in Nigeria with the country reporting the highest incidence and mortality globally. Recent national data suggests increasing incidence and expanding geographic spread. Predictors of LF case positivity in Nigeria have been sparsely studied. We thus sought to determine the sociodemographic and clinical determinants of LF positivity amongst suspected cases presenting to health facilities from 2018 to 2021. A secondary analysis of the national LF surveillance data between January 2018 and December 2021. Socio-demographic and clinical data of 20,027 suspected LF cases were analysed using frequencies and Chi-square statistics with significant p-value set at p < 0.05. The outcome variable was LF case status (positive or negative). Predictors of LF case positivity were assessed using multiple logistic regression models with 95% confidence intervals (CI). Case positivity rate (CPR) for the four years was 15.8% with higher odds of positivity among age group 40–49 years (aOR = 1.40; 95% CI 1.21–1.62), males (aOR = 1.11; 95% CI 1.03–1.20), those with formal education (aOR = 1.33; 95% CI 1.13–1.56), artisans (aOR = 1.70; 95% CI 1.28–2.27), religious leaders (aOR = 1.62; 95% CI 1.04–2.52), farmers (aOR = 1.48; 95% CI 1.21–1.81), and symptomatic individuals (aOR = 2.36; 95% CI 2.09–2.68). Being a health worker (aOR = 0.69; 95% CI 0.53–0.91), a teacher (aOR = 0.69; 95% CI 0.53–0.89) and cases reporting in the 3rd quarter (aOR = 0.79; 95% CI 0.69–0.92) had lower odds. In a sex-disaggregated analysis, female farmers had higher odds of positivity (aOR = 2.43; 95% CI 1.76–3.38; p < 0.001) than male farmers (aOR = 1.52; 95% CI 1.19–1.96; p < 0.01). Fever (aOR = 2.39; 95% CI 2.00–2.84) and gastrointestinal (GI) symptoms (aOR = 2.15; 95% CI 1.94–2.37) had the highest odds among symptoms. Combination of fever and GI symptoms (aOR = 2.15; 95% CI 1.50–3.10), fever and neurological symptoms (aOR = 6.37; 95% CI 1.49–27.16), fever and musculo-skeletal symptoms (aOR = 2.95; 95% CI 1.37–6.33), fever and cardiopulmonary symptoms (aOR = 1.81; 95% CI 1.24–2.64), and cardiopulmonary and general symptoms (aOR = 1.50; 95% CI 1.19–1.89) were also predictive. Cumulative LF CPR appears high with clearly identified predictors. Targeted interventions with heightened index of suspicion for sociodemographic categories predictive of LF in suspected cases are recommended. Ethnographic and further epidemiological studies could aid better understanding of these associations.

## Introduction

Lassa fever (LF) is a zoonotic viral disease that was first reported in northern Nigeria in 1969^1^. Since then, the virus has also been identified in several West African countries, including Sierra Leone, Guinea, Liberia, Ghana, Mali, and Benin Republic^2^. To date, many infections and associated deaths are being reported annually from the region^3^. In Nigeria, about 2787 confirmed cases of LF and 516 deaths were reported between December 2016 and September 2020^4^, while 630 confirmed cases and 112 deaths (case fatality rate (CFR) 17.8%) were recorded in the country as at epidemiologic week 10 of 2022^5^. Worse still, available data indicate that the virus is increasingly spreading beyond the traditional hotspots of Edo, Ondo, and Bauchi States, to other parts of the country^6^. Whereas only one geopolitical zone (GPZ) was affected in 2008, cases were detected in all six GPZs in 2018, and by 2021, 31 out of the 36 states and the Federal Capital Territory had reported confirmed cases^7,8^.

The prevention and control of LF in Nigeria are currently being implemented under two broad strategic frameworks: primary prevention and secondary prevention. Primary prevention consisting of environmental control measures, community engagement, contact tracing, depopulation of *Mastomys* rats responsible for the transmission of the virus, changes in farming practices, and the adoption of the “One Health” approach^9–11^ have been used in the country but with varying degrees of success. In contrast, secondary prevention premised on early diagnosis and prompt treatment of cases has had a higher rate of success. However, prevention of secondary transmission of LF is dependent on early presentation of symptomatic cases to the health facilities; rapid and accurate laboratory confirmation; and early isolation and institution of therapy. Low index of suspicion among healthcare workers (HCWs) due to the non-specific nature of symptoms promotes healthcare worker infections, especially with poor adherence to standard precautions. Furthermore, asymptomatic cases may not present to health facilities and could sustain transmission of the virus without detection. Poor health seeking behaviour in many settings and use of alternative healthcare could also lead to missed cases of LF in a health facility-based surveillance. Early presentation, detection, confirmation, isolation and treatment of cases coupled with strict adherence to infection prevention and control measures remain critical in the control of LF outbreak. The gold standard for LF diagnosis is the reverse transcription-polymerase chain reaction (RT-PCR)^12^. Less than a quarter of all suspected cases of LF may test positive to the virus by RT-PCR. Understanding predictors of case positivity among suspected cases of LF could improve index of suspicion among HCWs, facilitate early detection and isolation to limit spread, and guide prompt institution of therapy to improve outcomes and mitigate complications. To the best of our knowledge, there is paucity in literature of studies on the predictors of LF positivity in Nigeria; existing studies are descriptive and do not have the statistical power inherent in a large national surveillance data collected over a long period of time. Therefore, the objective of this study was to determine the sociodemographic, epidemiological, and clinical factors associated with LF positivity amongst suspected cases presenting to health facilities in Nigeria using 2018 to 2021 surveillance data.

## Methods

### Study design and setting

We conducted a retrospective analysis of LF surveillance data from health facilities in all the 36 states in Nigeria and the Federal Capital Territory (FCT), between January 2018 and December 2021.

### Data source

Medical record and health information of suspected Lassa cases and definitive diagnoses were entered into the Nigerian Surveillance Outbreak Response Management and Analysis System (SORMAS)^13^. SORMAS electronic health surveillance database served as the primary data source for this study. SORMAS is the primary digital platform for implementing the IDSR in Nigeria. This study used retrospective routine data on 20,027 suspected LF cases collected as part of LF surveillance in Nigeria from 2018 to 2021. The analysis excludes 35 probable cases recorded within the period.

### Data collection

The national routine surveillance for LF comprises data from laboratory, case management (clinical) and case investigation (epidemiological) forms. The forms capture demographic information (age, sex, residential address, occupation, level of education), date of admission, hospital name, clinical details (date of symptom onset, symptoms) and exposures (contact with known or suspected LF case, contact with rodents or rodent faeces and urine, participation in burial activity), and result of LF confirmatory test.

All suspected LF cases were investigated using a Lassa fever case investigation form (CIF). All suspected, probable, and confirmed cases were line-listed, and the information in the CIFs was uploaded real-time on SORMAS. Samples were collected and tested for all living suspected cases.

### Laboratory confirmation

Blood samples were collected from all patients suspected of having LF either in the inpatient or outpatient departments of a health facility. Neonates whose mothers had Lassa fever were tested regardless of whether they exhibited symptoms. Blood sample was triple packaged and aseptically transported in cold chain to any of the seven designated LF laboratories in Nigeria for confirmatory test by reverse transcriptase-polymerase chain reaction (RT-PCR).

### Definition of key study variables

#### Clinical variables

A total of 39 symptoms were recorded in the CIF data, but only 26 with at least one yes response were included in this analysis. The response options were yes, no, and unknown. Each symptom was recoded into a dummy variable where yes was coded 1 and no and unknown were coded 0. To minimise unstable estimates of effect from small samples, which was the case for most of the clinical variables, based on a consensus reached by the study clinicians, we merged individual clinical signs or symptoms associated with LF into composite variables, except fever which was reported in 88% of the symptomatic cases. The composition of each study variable is summarised in Supplementary file 1.

### Data management

#### Handling of missing data

De-identified data on LF were retrieved from NCDC SORMAS database. We adopted a complete-case approach to analysis of socio-demographic and other variables. All the variables included in this analysis were complete except sex with missing data (1.82%). We excluded variables with incomplete and incorrect data of up to 5% from the analysis to minimise loss of power.

### Statistical analysis

Demographic and clinical characteristics of the study participants in relation to the outcome variable were described in terms of frequencies and percentages (%), and chi-square to measure association. This enabled us to characterize the pattern of LF positivity by the demographic, time, exposure, and clinical variables.

To assess the association between individual covariate and outcome variables, we conducted bivariable logistic regression analyses for each explanatory variable and presented the findings as unadjusted odds ratios (ORs) and 95% Confidence Intervals (95% CIs). This was then followed by multivariable analysis using a multiple logistic regression. To explore possible gender dimensions to risks of LF, we fitted sex-disaggregated unadjusted and adjusted logistic regression models. To identify the significant clinical predictors of LF, we performed a sub-analysis with only symptomatic cases (N = 15,903) using the composite symptom variables. Three models were estimated, the unadjusted bivariate model for each symptom composite variable, a model 1 which contained all the symptoms variables, and model 2 which was a full model that contained all the symptom variables and adjusted for sociodemographic and time variables. To identify the combination of symptoms that predict LF positivity, we estimated several unadjusted and adjusted (with sociodemographic and time variables) regression models with two-way and three-way interaction of fever and each of the composite clinical variables, and between the composite clinical variables adjusting for fever. None of the three-way interactions was statistically significant. All the analyses were two-tailed and conducted with Stata 13 for windows.

### Ethics approval and consent to participate

The protocol for this study was reviewed and approved by the Nigeria National Health Research Ethics Committee (Approval Number: NHREC/01/01/2007-27/04/2022). The NCDC principles on ethical considerations (anonymity and confidentiality of records) in the conduct of research activities were strictly adhered to throughout the conduct of this study. All the information extracted was part of routine LF outbreak response data and activities. All data were kept confidential and stored in password embedded computers. Personal identifiers were not extracted, and dataset was de-identified prior to analysis. All methods were carried out in accordance with relevant guidelines and regulations.

## Results

### Socio-demographic characteristics of the Lassa fever suspected cases

The characteristics of the suspected cases (N = 20,027) are presented in Table 1. Males accounted for more of the suspected cases with 52.6%, and the median age was 34 (IQR 31) with 20–29 years and 60+ age groups accounting for 20.5% and 20.0% respectively. Edo state yielded nearly half of all suspected cases (49.4%). Over 86% of the suspected cases had attained formal education, and slightly above one-quarter were civil/public servants. More cases were reported in 2020 (38.7%) compared to other years, and 48.6% of cases were seen in the 1st quarter of the year representing the dry season in Nigeria. Over 79% of suspected cases were symptomatic. Of all suspected cases with contact history (1308), contact with a confirmed case (40.9%) or probable case (40.7%) were the most frequently reported (not shown in table). Only 18.4% of all suspected cases with contact history reported contact with rodents or rodents’ excreta.

**Table 1:** Association between sociodemographic factors and Case Positivity among Lassa fever suspected cases in Nigeria from 2018 to 2021.

Variable	Lassa fever Status (N = 20,027)		Chi-square	p-value
	Negative	Positive N (%)		
	N (%)	3162 (15.79)		
	16,865 (84.21)			
Sex (N = 19,662)			10.4559	0.001
Female	7918 (85.03)	1394 (14.97)		
Male	8626 (83.34)	1724 (16.66)		
Age group (in years)			90.6707	< 0.001
0–4	1082 (91.00)	107 (9.00)		
5–9	199 (88.05)	27 (11.95)		
10–19	2266 (85.48)	385 (14.52)		
20–29	3407 (82.92)	702 (17.08)		
30–39	3066 (83.41)	610 (16.59)		
40–49	1975 (80.61)	475 (19.39)		
50–59	1424 (82.74)	297 (17.26)		
60+	3446 (86.04)	559 (13.96)		
Educational level			64.2333	< 0.001
No formal education	2421 (89.43)	286 (10.57)		
Formal education	14,444 (83.39)	2878 (16.61)		
Occupation			141.3879	< 0.001
Artisan	216 (71.29)	87 (28.71)		
Child/pupil	2011 (88.55)	260 (11.45)		
Civil/public servant	3645 (82.69)	763 (17.31)		
Farming/livestock	768 (77.42)	224 (22.58)		
Health worker	573 (87.48)	82 (12.52)		
Religious leader	86 (74.14)	30 (25.86)		
Retiree	174 (82.08)	38 (17.92)		
Student	2681 (84.60)	488 (15.40)		
Teacher/lecturer	688 (87.53)	98 (12.47)		
Trader	2814 (84.38)	521 (15.62)		
Unemployed	2198 (85.89)	361 (14.11)		
Other	1011 (82.80)	210 (17.20)		
State of residence			10.4346	0.108
Other states	2112 (84.22)	387 (15.49)		
Edo	8337 (84.22)	1562 (15.78)		
Ondo	3901 (83.77)	756 (16.23)		
Ebonyi	1062 (86.41)	167 (16.59)		
Bauchi	731 (82.79)	152 (17.21)		
Plateau	378 (86.30)	609 (13.70)		
Taraba	344 (81.52)	78 (18.48)		
Time of report (in quarters)			36.8773	< 0.001
4th quarter	3297 (85.64)	553 (14.36)		
3rd quarter	2827 (86.88)	427 (13.12)		
2nd quarter	2677 (83.89)	514 (16.11)		
1st quarter	8064 (82.86)	1668 (17.14)		
Date of report (year)			129.4918	< 0.001
2018	1563 (80.15)	387 (19.85)		
2019	4818 (84.19)	905 (15.81)		
2020	6371 (82.31)	1369 (17.69)		
2021	4113 (89.14)	501 (10.86)		
Symptom status			139.1280	< 0.001
Asymptomatic	3719 (90.18)	405 (9.82)		
Symptomatic	13,146 (82.66)	2757 (17.34)		

### Case positivity rate

The case positivity rate (CPR) for the various sub-groups and the corresponding chi-square test result are presented in Table 1. Cumulative CPR for the four years was 15.8% (Table 1), ranging from 10.9% in 2021 to 19.9% in 2018. CPR was higher among males, and among suspected cases aged 20 to 59 with the highest positivity rate among the age group 40–49 years. A higher CPR was observed among those with formal education. Compared to other occupational groups, CPR among suspected cases in farming/livestock occupation, artisans, and religious leaders was over 20% with the highest among artisans (28.7%). The CPR in the six hotspot States ranged from 13.7 to 18.5% with the highest rate in Taraba State. CPR was higher in the first and second quarters of the year. The CPR among symptomatic cases was nearly double that among asymptomatic cases. All the observed differences in CPR except for State of residence were statistically significant.

### Multivariable analysis

The result of the logistic regression models predicting the socio-demographic determinants of LF case positivity is presented in Table 2. Age significantly predicted case positivity for suspected cases aged 20 to 59 compared to older cases aged 60 and over. Children aged 0–4 years were 40% less likely to be LF positive only in the unadjusted model. The odds of case positivity were 11% higher for males compared to females. Suspected cases who had formal education were 33% more likely to have positive LF test result than those without. Occupational groups predicted the odds of LF case positivity. Holding other variables constant in the adjusted model, the likelihood of LF case positivity among artisans, farmers (crop and livestock), and religious leaders was 70%, 48% and 62% more, respectively, compared to suspected cases who were unemployed. In contrast, the likelihood of case positivity was significantly lower for children/pupils (AOR 0.82; 95% CI 0.68–0.99), health workers (AOR 0.69; 95% CI 0.53–0.91), and teachers (AOR 0.69; 95% CI 0.53–0.89). LF case positivity was higher in the 1st and 2nd quarters of the year, but when other factors were controlled, the odds were 12% higher in the 1st quarter, and 21% lower in the 3rd quarter compared to the 4th quarter of the year. Compared to 2018, the odds of LF case positivity were significantly lower in 2019, 2020 and 2021. Symptomatic suspects were 2.36 times as likely as the asymptomatic suspected cases to be LF positive holding other factors constant (95% CI: 2.09–2.68).

**Table 2:** Logistic regression models of the socio-demographic predictors of Lassa fever positivity in Nigeria.

Variable	Unadjusted model	Adjusted model
	OR (95% CI)	AOR (95% CI)
Age		
60+ (Ref)		
0–4	0.60 (0.49–0.75)***	0.86 (0.67–1.09)
5–9	0.83 (0.55–1.26)	0.87 (0.57–1.32)
10–19	1.04 (0.91–1.20)	1.11 (0.95–1.30)
20–29	1.27 (1.1201.43)***	1.27 (1.11–1.45)***
30–39	1.22 (1.08–1.38)**	1.19 (1.04–1.37)**
40–49	1.48 (1.29–1.69)***	1.40 (1.21–1.62)***
50–59	1.28 (1.10–1.49)**	1.20 (1.02–1.41)*
Sex		
Female (Ref)		
Male	1.13 (1.05–1.22)**	1.11 (1.03–1.20)**
Education		
No formal education (Ref)		
Formal education	1.68 (1.48–1.91)***	1.33 (1.13–1.56)***
Occupation		
Unemployed (Ref)		
Artisan	2.45 (1.86–3.22)***	1.70 (1.28–2.27)***
Child/pupil	0.78 (0.66–0.93)**	0.82 (0.68–0.99)*
Civil/public servant	1.27 (1.11–1.46)***	1.12 (0.96–1.31)
Farming/livestock	1.77 (1.47–2.13)***	1.48 (1.21–1.81)***
Health worker	0.87 (0.67–1.12)	0.69 (0.53–0.91)**
Religious leader	2.12 (1.38–3.26)**	1.62 (1.04–2.52)*
Retiree	1.32 (0.92–1.92)	1.44 (0.98–2.14)
Student	1.10 (0.95–1.28)	0.97 (0.81–1.15)
Teacher/lecturer	0.86 (0.68–1.10)	0.69 (0.53–0.89)**
Trader	1.12 (0.97–1.30)	1.09 (0.92–1.28)
Other	1.26 (1.05–1.52)*	1.10 (0.90–1.35)
State of residence		
Other states (Ref)		
Edo	1.02 (0.90–1.15)	0.98 (0.87–1.11)
Ondo	1.05 (0.92–1.20)	1.07 (0.93–1.22)
Ebonyi	0.85 (0.70–1.04)	0.93 (0.76–1.13)
Bauchi	1.13 (0.92–1.39)	1.11 (0.90–1.38)
Plateau	0.86 (0.64–1.16)	0.90 (0.67–1.22)
Taraba	1.23 (0.94–1.61)	1.24 (0.94–1.63)
Time of report (in quarters)		
4th quarter (ref)		
3rd quarter	0.90 (0.78–1.03)	0.79 (0.69–0.92)**
2nd quarter	1.14 (1.00–1.30)*	0.98 (0.85–1.12)
1st quarter	1.23 (1.11–1.36)***	1.12 (1.00–1.25)*
Date of report (year)		
2018 (Ref)		
2019	0.75 (0.66–0.86)***	0.59 (0.51–0.69)***
2020	0.86 (0.76–0.98)*	0.64 (0.56–0.75)***
2021	0.49 (0.42–0.56)***	0.34 (0.29–0.40)***
Symptom status		
Asymptomatic (Ref)		
Symptomatic	1.92 (1.72–2.15)***	2.36 (2.09–2.68)***

***p < 0.001; **p < 0.01; *p < 0.05.

### Sex-specific analysis

**Figure 1:**
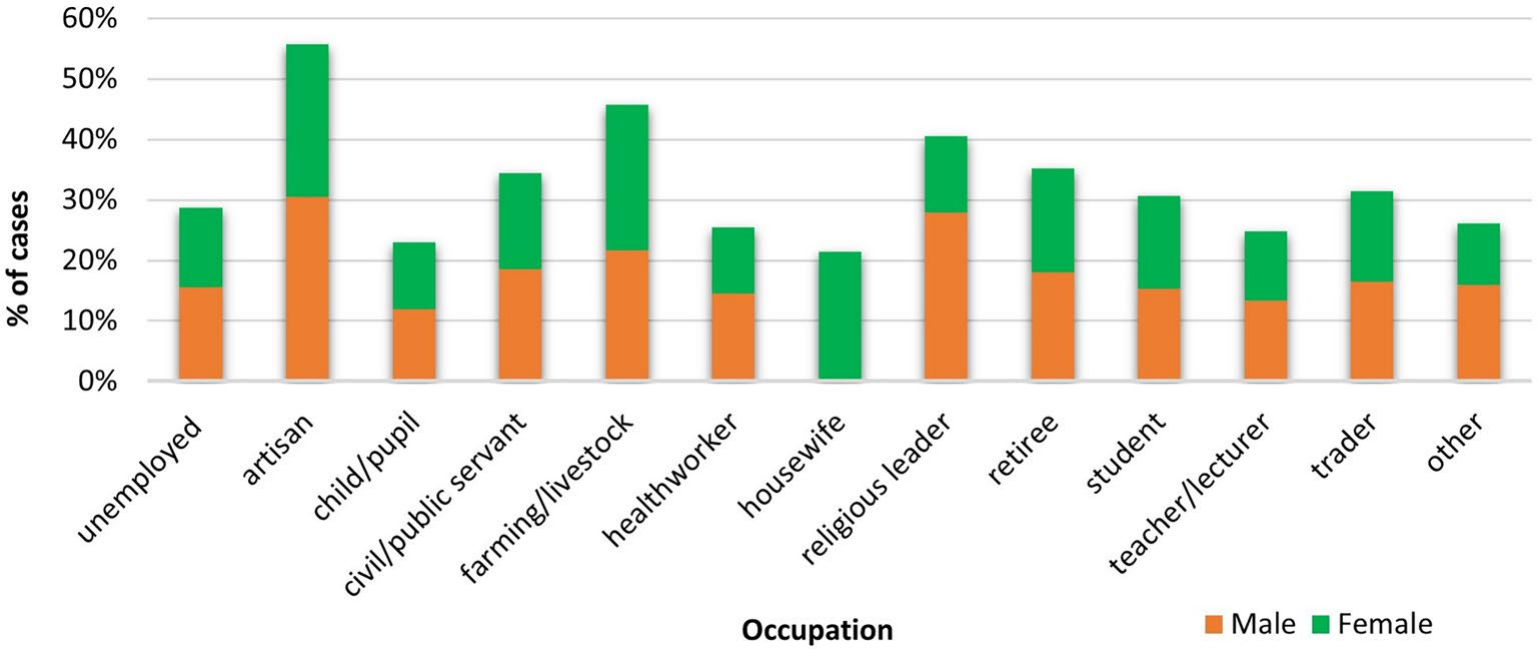
Sex-disaggregated LF positivity by occupational categories in Nigeria, 2018–2021.

To explore a possible gender dimension to LF case positivity in Nigeria, we estimated sex-disaggregated models of socio-demographic predictors (Table 3). In contrast to women, age was a significant predictor of LF case positivity for men, particularly for ages 20–49 years. Both male and female children aged 0–4 years were less likely to be LF positive, but this was only statistically significant in the unadjusted models. Suspected cases who attained formal education were more likely to be LF positive compared to those who had no formal education, but this was only significant for men (AOR 1.48; 95% CI: 1.18 to 1.86). Women and men who were artisans were over twice as likely to be LF positive as suspected cases who were unemployed. Farming and livestock occupations exposed both women and men to higher odds of being LF positive than the unemployed, but the odds were 2.43 times as high for women compared to 1.52 times for men. Significant sex-related differences in other occupations were observed (Fig. 1). For women, being a civil/public servant increased the odds of LF positivity by 37%, being a housewife by 91%, a student by 40% and a trader by 41%. For men, being a religious leader (AOR 2.01; 95% CI 1.25–3.23) and retiree (AOR 1.71; 95% CI 1.04–2.80) increased the odds of LF positivity but being a teacher/lecturer was protective (AOR 0.70 CI 0.50–0.99).

**Table 3:** Sex-disaggregated logistic regression models predicting Lassa fever case positivity.

Characteristic	Female		Male	
	OR (95% CI)	AOR (95% CI)	OR (95% CI)	AOR (95% CI)
Age				
60+ (Ref)				
0–4	0.61 (0.44–0.85)**	0.80 (0.55–1.14)	0.59 (0.44–0.80)**	0.83 (0.60–1.16)
5–9	1.06 (0.61–1.84)	1.05 (0.60–1.84)	0.63 (0.33–1.19)	0.60 (0.31–1.15)
10–19	0.96 (0.77–1.18)	0.97 (0.78–1.22)	1.09 (0.90–1.32)	1.11 (0.90–1.37)
20–29	1.13 (0.94–1.35)	1.10 (0.91–1.33)	1.38 (1.16–1.63)***	1.34 (1.11–1.60)**
30–39	1.01 (0.84–1.22)	0.98 (0.80–1.19)	1.39 (1.17–1.66)***	1.35 (1.12–1.62)**
40–49	1.17 (0.95–1.44)	1.15 (0.92–1.43)	1.74 (1.45–2.08)***	1.62 (1.34–1.96)***
50–59	1.23 (0.98–1.54)	1.19 (0.94–1.51)	1.26 (1.02–1.56)*	1.19 (0.95–1.48)
Educational level				
No formal education (Ref)				
Formal education	1.45 (1.19–1.76)***	1.17 (0.93–1.47)	1.89 (1.56–2.27)***	1.48 (1.18–1.86)**
Occupation				
Unemployed (Ref)				
Artisan	2.23 (1.41–3.53)**	2.33 (1.45–3.75)***	2.36 (1.67–3.34)***	2.03 (1.42–2.90)***
Child/pupil	0.81 (0.62–1.06)	1.05 (0.78–1.41)	0.73 (0.58–0.92)**	0.99 (0.77–1.27)
Civil/public servant	1.25 (1.00–1.56)*	1.37 (1.07–1.75)*	1.22 (1.02–1.47)*	1.16 (0.95–1.43)
Farming/livestock	2.10 (1.54–2.87)***	2.43 (1.76–3.38)***	1.49 (1.17–1.89)**	1.52 (1.19–1.96)**
Health worker	0.80 (0.55–1.18)	0.86 (0.58–1.28)	0.92 (0.64–1.31)	0.86 (0.59–1.24)
Housewife	1.81 (1.36–2.40)***	1.91 (1.42–2.58)***	–	–
Religious leader	0.94 (0.21–4.21)	0.96 (0.21–4.31)	2.10 (1.32–3.33)**	2.01 (1.25–3.23)**
Retiree	1.36 (0.73–2.55)	1.77 (0.92–3.37)	1.18 (0.74–1.89)	1.71 (1.04–2.80)*
Student	1.19 (0.94–1.51)	1.40 (1.08–1.83)*	0.98 (0.80–1.19)	1.12 (0.89–1.40)
Teacher/lecturer	0.85 (0.58–1.24)	0.83 (0.56–1.24)	0.83 (0.60–1.14)	0.70 (0.50–0.99)*
Trader	1.16 (0.93–1.46)	1.41 (1.10–1.82)**	1.06 (0.87–1.29)	1.24 (0.99–1.54)
Other	0.74 (0.48–1.16)	0.78 (0.49–1.23)	1.02 (0.76–1.38)	1.02 (0.75–1.39)

Season, year of report, and state of residence were controlled in the adjusted models.

***p < 0.001; **p < 0.01; *p < 0.05.

### Clinical symptoms and signs of Lassa fever

**Figure 2:**
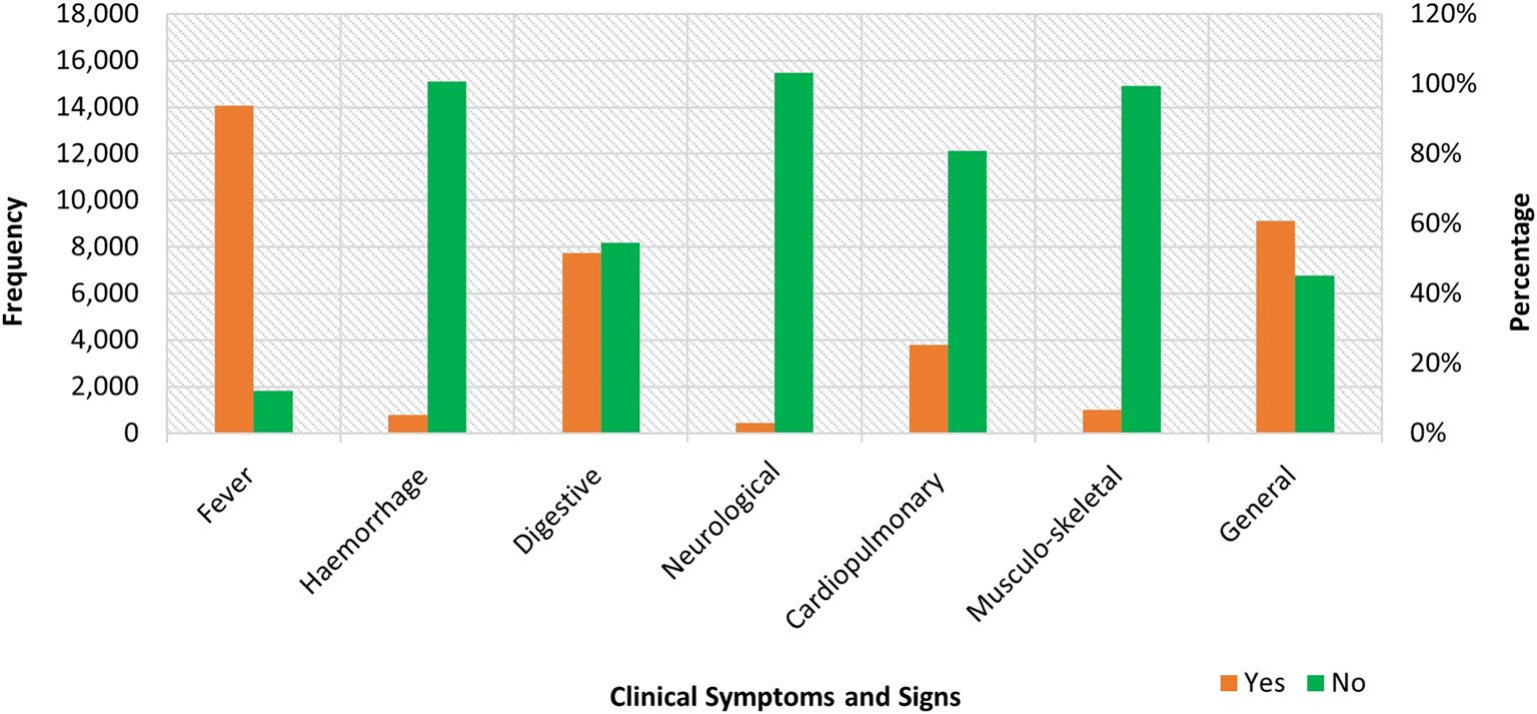
Clinical characteristics of lassa fever suspected cases in Nigeria, 2018–2021.

**Figure 3:**
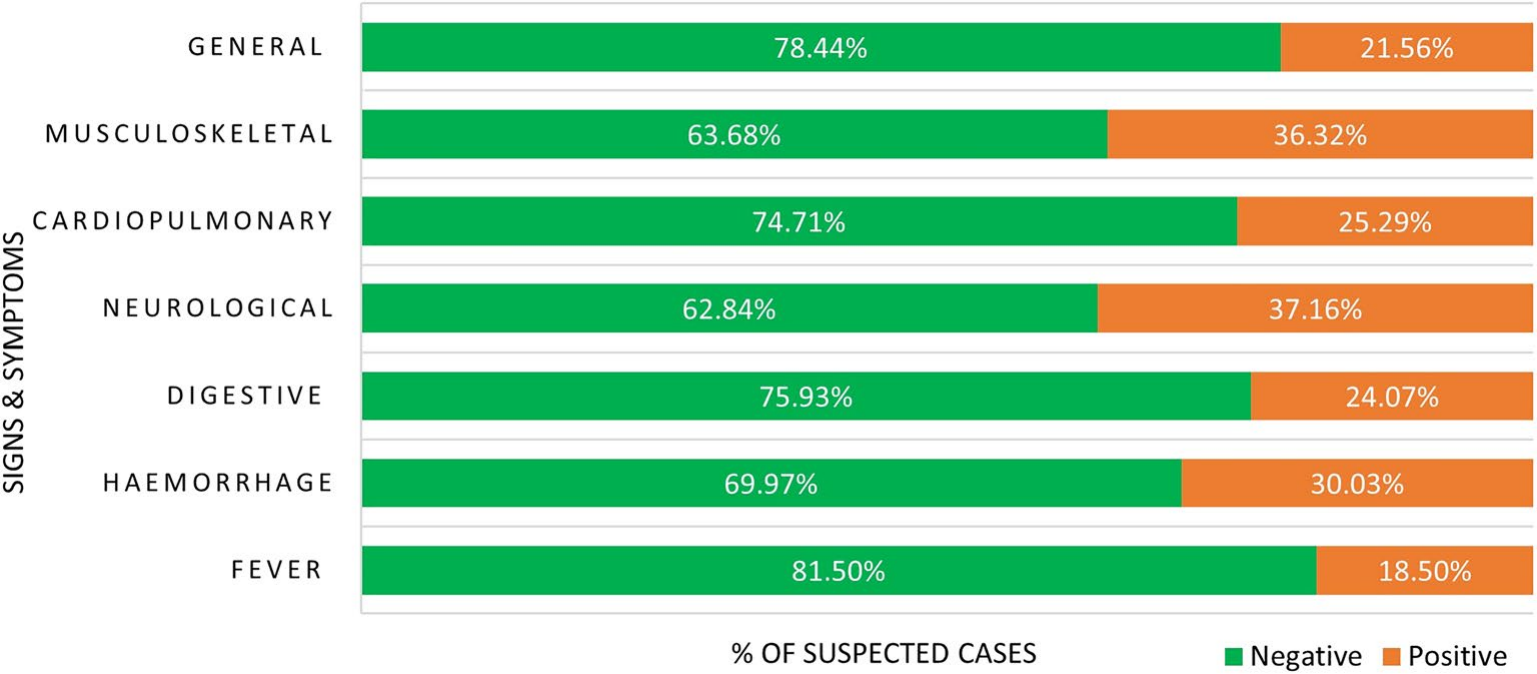
Lassa fever positivity status by symptoms and/or signs among suspected cases in Nigeria, 2018–2021.

The results of the sub-analysis for clinical symptoms and signs of LF positivity are presented in Figs. 2 and 3, and Table 4. The most reported symptoms are fever (88.5%), general symptoms (57.4%), Digestive/gastrointestinal symptoms (48.6%), and cardiopulmonary symptoms (23.8%); and the least reported were neurological symptoms (2.7%) (Fig. 2).

**Table 4:** Logistic regression models predicting case positivity by clinical symptoms and signs among suspected Lassa fever cases in Nigeria 2018–2021.

Symptom	Unadjusted model	Adjusted model 1	Adjusted model 2
	OR (95% CI)	AOR (95% CI)	AOR (95% CI)
Fever			
No (Ref)			
Yes	2.47 (2.08–2.93)***	2.58 (2.17–3.07)***	2.39 (2.00–2.84)***
Haemorrhagic symptoms			
No (Ref)			
Yes	2.14 (1.83–2.51)***	1.39 (1.17–1.66)***	1.28 (1.07–1.54)**
Gastrointestinal & digestive			
No (Ref)			
Yes	2.57 (2.35–2.80)***	2.05 (1.86–2.25)***	2.15 (1.94–2.37)***
Central nervous system/neurological			
No (Ref)			
Yes	2.93 (2.40–3.57)***	1.56 (1.25–1.95)***	1.38 (1.09–1.74)**
Cardiopulmonary			
No (Ref)			
Yes	1.94 (1.77–2.12)***	1.31 (1.19–1.45)***	1.48 (1.34–1.64)***
Musculo-skeletal			
No (Ref)			
Yes	2.98 (2.60–3.41)***	1.90 (1.63–2.20)***	1.66 (1.42–1.93)***
General symptoms and signs			
No (Ref)			
Yes	2.08 (1.90–2.27)***	1.52 (1.38–1.67)***	1.68 (1.51–1.85)***
Interaction models			
Fever + haemorrhagic symptoms	1.24 (0.68–2.25)		1.39 (0.76–2.55)
Fever + gastrointestinal/digestive	1.91 (1.33–2.73)***		2.15 (1.50–3.10)***
Fever + CNS/neurological	6.72 (1.59–28.41)*		6.37 (1.49–27.16)*
Fever + cardiopulmonary	1.80 (1.24–2.61)**		1.81 (1.24–2.64)**
Fever + musculo-skeletal	3.21 (1.51–6.85)**		2.95 (1.37–6.33)**
Fever + general symptoms	1.37 (0.96–1.97)		1.32 (0.92–1.90)
Cardiopulmonary + haemorrhagic symptoms	1.59 (1.15–2.21)**		1.35 (0.96–1.89)†
General symptoms + haemorrhagic symptoms	1.72 (1.15–2.56)**		1.50 (0.99–2.25)^†^
Cardiopulmonary + general symptoms	1.67 (1.34–2.08)***		1.50 (1.19–1.89)***
Gastrointestinal/digestive + general symptoms	1.46 (1.21–1.77)***		1.17 (0.96–1.43)
CNS/neurological + general symptoms	1.89 (1.11–3.21)*		1.63 (0.94–2.84)^†^
Musculo-skeletal + general symptoms	1.57 (1.05–2.35)*		1.40 (0.92–2.12)
Gastrointestinal/digestive + musculo-skeletal	1.95 (1.34–2.84)***		1.44 (0.98–2.11)^†^
Gastrointestinal/digestive + cardiopulmonary	1.38 (1.10–1.73)**		1.20 (0.95–1.52)^†^
Cardiopulmonary + musculo-skeletal	1.65 (1.24–2.19)**		1.34 (1.00–1.81)*

Model 1 adjusted only the symptoms; model 2 adjusted the symptoms and the socio-demographic characteristics of the cases, and time variables.

Other interactions were neither statistically significant in both the unadjusted and adjusted models.

***p < 0.001; **p < 0.01; *p < 0.05.

^†^The adjusted models reached statistical significance (p < 0.05) when fever is not controlled.

All the composite symptoms were significantly associated with LF status. CPR ranged from 18.5% to 37.2% (Fig. 3). CPR was highest in suspected cases who presented with neurological symptoms and signs (37.2%; p < 0.001), musculoskeletal symptoms (36.3%; p < 0.001), and haemorrhage (30.0%; p < 0.001). It was lowest for fever (18.6%; p < 0.001).

We estimated several unadjusted and adjusted regression models to determine the independent relationship between the symptoms and LF case positivity (Table 4). In the unadjusted and adjusted models, the presence of all the composite symptoms was significantly related to LF case positivity. The largest adjusted odds were in fever and gastrointestinal/digestive symptoms. Individual Lassa fever suspected cases presented more than one symptom; thus, we conducted several two-way interaction models to determine the mix of symptoms that would be predictive of Lassa fever. All the unadjusted models between fever and the other symptoms were statistically significant except the model with fever and general symptoms. When other factors were adjusted (Model 2), the interaction terms were still significantly predictive of LF positivity except fever and general symptoms.

All the possible two-way interactions between the other symptoms (adjusting for fever and other variables) were conducted, only the models that presented some statistically significant results are presented. Compared to suspected cases who presented with none of the two interacted composite symptoms, the odds of LF positivity were 50% higher when suspected cases presented with cardiopulmonary and general symptoms, and 34% higher for cardiopulmonary and musculoskeletal symptoms. The other combinations of haemorrhagic and cardiopulmonary symptoms, haemorrhagic and general symptoms, neurological and general symptoms, digestive and musculoskeletal symptoms, and digestive and cardiopulmonary symptoms reached statistical significance when the effect of fever was not controlled. Gastrointestinal/digestive and general symptoms, and musculoskeletal and general symptoms were not predictive of LF positivity when other factors and fever were held constant.

## Discussion

The main objective of this study was to determine the sociodemographic, epidemiological, and clinical factors associated with LF diagnosis in suspected cases presenting to different health facilities in Nigeria over a 4-year period. Our study is one of the few attempts to use national surveillance data to predict LF test positivity. Case positivity rate was higher among suspected cases aged 20–59 in contrast with younger persons and those aged 60 and above. A similar result was found in a previous study in one State in Nigeria^14^. This pattern of LF positivity may partly be because more adults than children in Nigeria are exposed to contact with confirmed or probable LF cases, and conditions that increase susceptibility to LF. For instance, although child labour is still prevalent in Nigeria, the percentage of children ages 5–17 in hazardous work continues to decline owing to initiatives to eliminate child labour in the country^15,16^. In the multivariable analysis, age was a strong predictor of LF positivity for suspected cases who were aged 20–59 years. Of note is that this age effect was only significant for males in the sex-disaggregated analysis. However, this result highlights variation in exposure to infectious diseases by age and the importance of a life-course perspective in understanding the epidemiology of LF in Nigeria^17^. In a past study in Nigeria, age predicted LF case fatality^16^.

There was male preponderance in Lassa fever confirmed cases in our study. This aligns with the findings in a study of 1893 suspected cases of LF in Nigeria in 2018 that reported positivity rate of 68% in males and 34% in females^19^. The sex differential may be explained by gender roles. Males may be more occupationally exposed in the LF endemic settings. Artisanship, farming, and hunting are male-dominated occupations in Nigeria that could increase the risk of exposure to LASV. However, in the sex-disaggregated analysis, the odds of LF positivity were significantly high for both males and females in artisanship and farming occupations, with higher odds for females. This result however contradicts the finding of no sex association with LF positivity among 440 suspected cases admitted in a health facility in Ebonyi State^20^. The much lower sample size limited to one state in the latter study compared to the 20,027 from all states in our study may have lowered the power for demonstrating any significant sex difference.

Those with any form of formal education were about 33% more likely to test positive for LF than those with no formal education. While this may seem to deviate from the usual disease vulnerability of the uneducated, there are possible explanations. Those with formal education are more likely to dwell in urban areas^21^. Higher population density, housing deficit, poverty, and poor sanitation in urban areas in Nigeria^22^ could predispose to higher transmission rates of Lassa fever among the educated category. While Ogbu and colleagues^3^ described living in rural area as a risk factor for Lassa fever, they also identified crowded living conditions (more applicable to many urban settings in Nigeria) as another risk factor. Conversely, health seeking behaviour with presentation at health facilities could likely be commoner among those with formal education compared to those without^23,24^. This could be the reason for the lower number of suspected cases among the uneducated with possibility of missed cases. Strategies to improve health-seeking behaviour among the uneducated, such as the use of indigenous languages and non-literary channels of risk communication, are recommended especially in rural areas. In-depth anthropological enquiries into the social and behavioural risk factors for Lassa fever positivity among those with any level of formal education are also required.

Surprisingly, State of residence was not a significant determinant of positivity in this study. Positivity rate in the high burden states^19^ did not differ significantly from the rate in other states. While this could be a pooled effect from merging of the low burden states during analysis, it could reflect ongoing unreported transmissions in the ‘silent’ states. This makes the case for surveillance system evaluation, particularly targeting the ‘silent’ states. With subclinical presentation in about 80% of cases^25^, vague presentation of symptoms, and poor health-seeking behaviour in many Nigerian communities^26^, it is likely that states with little or no reported cases may be harbouring the disease. A nationwide community-based seroprevalence survey could help determine the actual burden of Lassa fever in Nigeria. Furthermore, integrating community-based surveillance with the existing health facility-based surveillance could make the system more sensitive in picking up cases.

Contact with either a confirmed or probable case was the most frequently reported contact type among confirmed cases of LF in Nigeria. Whereas contact with rodents or rodents excreta was the least reported, this is a documented route of primary transmission of LASV^3,25^. Eliciting information on rodent contact may be difficult due to recall bias. Exposure to rodent excreta could happen unwittingly and these may have been wrongly classified in the responses. Nevertheless, the study findings suggest significant human-to-human transmission of LASV in Nigeria. Community sensitisation on contact precautions and promotion of hygienic practices should be sustained to control human-to-human transmissions of LF.

While artisans, farmers, and religious leaders had higher odds of testing positive for LF compared to the unemployed, healthcare workers, children/pupils and teachers/lecturers were less likely to test positive. The higher risk of LF positivity among artisans may not be unexpected, though it could be difficult to ascertain its specific predictiveness. Artisanship includes a heterogeneous mix of trade, some of which might involve the use of animal and animal products in crafts. Artisans that also engage in farming are more likely to report the former as their occupation; same as other occupational categories that are also engaged in farming activities. Clustering of artisans of similar trade could increase population density in their work environment with a higher risk of exposure of food to rodents. Further scrutiny of this group to identify specific work groups could provide a better insight into the occupational risk points. Occupation as a risk factor may be difficult to elicit using routine surveillance data due to multiple occupational categories that can apply to the same individual. One of the first cases of LF recorded in Ghana in 2011 was a 19-year-old farmer, hunter, miner, and trader in wild animals^27^. Nonetheless, occupations that increase the chances of human contact with rodents, their body fluids, or infected humans could increase the risk of transmission of the virus without adequate infection prevention and control measures^19^. Young children understandably could be protected from occupational exposure to the reservoirs of LF. The apparent lower likelihood to test positive amongst health workers needs to be further examined but may be due to better awareness, a higher index of suspicion, availability and use of personal protective equipment with better hygiene practices, and also a lower threshold for testing.

The sex-disaggregated analysis reveals gendered occupational exposure to LASV. In a male-dominated occupation such as the clergy, men’s exposure to LF positivity was significantly high, but not for women. Female-dominated occupations such as being a housewife exposed women to LF, as well as trading in contrast with men. Compared to 26% of men, 62% of women in Nigeria work in the informal sales/service sector comprising mainly of petty trading^19^. Furthermore, female farmers had higher odds of LF positivity than their male counterparts in this study. These findings are suggestive of the critical role of gender as a social determinant of health outcomes^28^. The gender-based occupational vulnerability requires further scrutiny to address inequity in work or role-based safety. It is also suggestive of a need to incorporate gender-transformative strategies in LF fever research, control and elimination programmes in Nigeria.

LF positivity was highest during the first quarter of the year corresponding to the peak of the dry season. In Sierra Leone, a similar peaking of confirmed cases of Lassa fever during the dry season has also been described^29^. Dry windy weather promotes the spread of spores and fomites thereby driving transmission. Farming practices such as bush burning and deforestation during the dry season displace rodents from their natural habitats and increase the chances of infiltration of residential areas by rodents, hence increasing the animal-human interface^30^. Dry seasons also represent the harvest period in many communities with processing of grains near residential areas attracting rodents to people’s houses.

We examined the symptoms of LF presented by patients (excluding the clinical complications). When the effect of other factors was adjusted, all the individual composite symptoms and signs were independently predictive of LF positivity. The most predictive were fever and gastrointestinal/digestive symptoms. This result is supported by a systematic review of 147 previous studies that identified the most commonly presented symptoms of LF^31^. LF presents with a wide spectrum of symptoms and signs, some of which are not specific to LF, and sometimes distinctively different signs. Therefore, we sought to identify the mix of symptoms and signs that significantly predict LF. Our findings show that the odds of LF are high in patients who present with a mix of fever and any of the gastrointestinal/digestive, cardiopulmonary, central nervous system/neurological, and musculoskeletal symptoms. Furthermore, without fever, a combination of cardiopulmonary and musculoskeletal symptoms, and cardiopulmonary and general symptoms are significantly predictive of LF. Paradoxically, a combination of fever and haemorrhagic symptoms was not a significant predictor of Lassa fever. This might be the result of a constellation of so many diagnoses like septicaemia with disseminated intravascular coagulation, thrombocytopenias, other viral haemorrhagic fevers not routinely screened for, or diseases like chronic liver disease that could be complicated by bleeding and spontaneous bacterial peritonitis. These results provide a useful guide for a quick diagnosis of LF. Timely and accurate diagnosis and treatment are essential for preventing mortality and making sustained progress towards the control and elimination of LF in a limited-resource country such as Nigeria. A case–control study in Sierra Leone supports some of our findings. The study found a combination of fever, pharyngitis, retrosternal pain, and proteinuria were predictive of LF^32^.

To the best of our knowledge, there is paucity of studies that go beyond the descriptive identification of specific symptoms and signs of LF. Most studies that attempt to identify a combination of symptoms and signs using predictive models focus on LF mortality and other outcomes^18,19,33^.

### Study strengths and limitations

To the best of our knowledge, this is the first study that draws data from all the Nigerian States to predict the socio-demographic, exposure and clinical predictors of LF positivity. Other studies are either descriptive or sub-national. However, this study has some limitations. Although the data were drawn from the entire country, they are based on facility-based surveillance, only those who present in the health facilities are included. Thus, the data are not representative of the entire population. Also, there are the problems of under-reporting, incomplete and inaccurate data, and changes in case definition over time often associated with surveillance data. However, Nigeria has maintained a specific definition of suspected cases since 2018. Missing data in sex was less than 1% which could not have biased the result, but we dropped a few variables in the analysis due to a large number of incomplete reporting which cannot be completed using statistical procedures.

## Conclusion

Cumulative CPR for LF in Nigeria from 2018 to 2021 was 15.9%. Yearly CPR ranged from 10.9% in 2021 to 19.9% in 2018. Age, sex, occupation, education, symptom categories and time of the year of reporting were independently associated with LF positivity. Public health interventions targeting the vulnerable groups from this study are recommended for effective prevention and control of LF in Nigeria. A heightened index of suspicion among clinicians for cases presenting with symptom groupings described as predictive in this study even in the absence of fever could improve early detection of LF and reduce missed cases. Ethnographic and further epidemiological studies of occupational and gender-based risk factors of LF are recommended to provide a more in-depth understanding of structural, social and societal determinants of LF infection in Nigeria.

## Supplementary Information

Supplementary Information.

## Data Availability

The dataset for this study is available from the Nigeria Centre for Disease Control on reasonable request from the corresponding author.
