# Supplementary material for: Predictors of Lassa fever diagnosis in suspected cases reporting to health facilities in Nigeria
Source: Sci Rep. 2023 Apr 21;13:6545. doi: 10.1038/s41598-023-33187-y (PMC10121657; doi:10.1038/s41598-023-33187-y)
Supplement: Supplementary file 1 — Supplementary Information. [file 41598_2023_33187_MOESM1_ESM.docx]

**Predictors of Lassa fever diagnosis in suspected cases reporting to health facilities: A secondary data analysis of Nigerian surveillance data, 2018-2021**

**Definition of Variables**

| **Variable** | **Definition** |
| --- | --- |
| **Concepts** |  |
| Suspected case of Lassa fever | Any individual presenting with one or more of the following: malaise, fever, headache, sore throat, cough, nausea, vomiting, diarrhoea, myalgia, chest pain, hearing loss and either: (a) history of contact with excreta or urine of rodents;(b) history of contact with a probable or confirmed Lassa fever case within a period of 21 days of onset of symptoms or any person with inexplicable bleeding/haemorrhage from January 2018 in the State. |
| Confirmed case of Lassa fever | Any suspected case with laboratory confirmation (positive IgM antibody, PCR or virus isolation). RT-PCR was used in the diagnosis for all suspected cases. |
| Case positivity rate | The case positivity rate (CPR) was the number of suspected cases in a sub-group who tested positive divided by the total number of suspected cases in that group multiplied by 100. |
| Probable case of Lassa fever | Any suspected case who died or absconded without collection of specimens for laboratory testing. |
| Non-case of Lassa fever | A suspected case whose RT-PCR test was negative for Lassa virus. |
| Contact | A person who has been exposed to an infected person, or to an infected person’s secretions, excretions, or tissues within three weeks of last contact with a confirmed or probable case of Lassa fever. For this study, we have included contact with rodent or its body fluids in the contact history for analysis. |
| Healthcare workers | All personnel working in health facilities regardless of whether they are in core clinical services, supportive functions or in training. |
| **Outcome variable** | |
| Lassa fever status | Lassa fever case status categorised as positive or negative by RT-PCR test. |
| **Explanatory variables** | |
| Age | Originally a continuous variable but transformed into categories as 0-4, 5-9, 10-19, 20-29, 30-39, 40-49, 50-59, and 60+ to capture common social exposures by age groupings. |
| Sex | Binary category of either male or female based on self-report. |
| Educational Level | A categorical variable in line with the Nigerian educational system: no formal education; nursery/primary; secondary; and tertiary. All except ‘no formal education’ were grouped as ‘formal education’ and the dichotomous variable, ‘no formal education; formal education’ used for further analysis. |
| State of residence | Place of residence of a suspected case at the time of presentation at health facility consisting of the 36 states and the federal capital territory (FCT). Based on number of cases reported routinely, the six hotspot states were treated as standalone categories while the rest were classified as ‘others’. |
| Occupation | Source of income of patient at time of presentation categorized as artisan; child/pupil; civil/public servant; farming/livestock; housewife; trader/business; health worker; religious leader; student; teacher/lecturer; retiree; and other. |
| Season | Based on previously reported seasonality of Lassa fever in Nigeria, this variable was categorised into quarters of the year: January-March (first quarter); April-June (second quarter); July-September (third quarter); and October-December (fourth quarter). |
| Clinical variables | Individual symptoms and signs and their further groupings into systems and combination of systems for further analysis of predictiveness. |
